# Plasma TDP-43 is a potential biomarker for advanced limbic-predominant age-related TDP-43 encephalopathy neuropathologic change

**DOI:** 10.1186/s13024-025-00910-4

**Published:** 2025-11-14

**Authors:** Jijing Wang, Julie A. Schneider, David A. Bennett, Nicholas T. Seyfried, Tracy L. Young-Pearse, Hyun-Sik Yang

**Affiliations:** 1 Department of Neurology, Brigham and Women’s Hospital, Boston, MA 02115 USA; 2 Harvard Medical School, Boston, MA 02115 USA; 3 The Broad Institute of MIT and Harvard, Cambridge, MA 02142 USA; 4 Rush Alzheimer’s Disease Center, Rush University Medical Center, Chicago, IL 60612 USA; 5 Department of Neurological Sciences, Rush University Medical Center, Chicago, IL 60612 USA; 6 Department of Biochemistry, Emory University School of Medicine, Atlanta, GA 30322 USA

## To the Editor

Limbic-predominant age-related TDP-43 encephalopathy neuropathologic change (LATE-NC) is a major cause of late-onset amnestic dementia, yet no diagnostic biomarker is available [1]. The recently published clinical criteria operationally defined “probable LATE” in older individuals with negative Alzheimer’s disease (AD) biomarkers [2]. However, most LATE-NC cases are comorbid with Alzheimer’s disease neuropathologic change (ADNC), and in vivo diagnosis of LATE-NC remains challenging without a specific molecular biomarker [2]. Prior plasma biomarker investigations of other TDP-43 proteinopathies, such as frontotemporal lobar degeneration or amyotrophic lateral sclerosis (ALS), using various TDP-43 immunoassays have shown conflicting results [3]. Measuring peripheral extracellular vesicles (EV) TDP-43 has shown initial promise [4], but EV-based methods are currently limited. Recently, plasma phospho-TDP-43 (pTDP-43) measured with a highly sensitive Nucleic Acid Linked Immuno-Sandwich Assay (NULISA) has been shown to be elevated in ALS [5], and another study showed an association between NULISA-measured plasma pTDP-43 with findings suggestive of underlying LATE-NC (hippocampal atrophy and cognitive decline) [6]. Here, we examine the biomarker potential of plasma TDP-43 measured with NULISA in detecting advanced LATE-NC.

We analyzed the data from fifty deceased participants from an extensively characterized clinical-pathologic cohort—the Religious Orders Study and the Rush Memory and Aging Project [7] (ROSMAP; *N* = 18 no/low ADNC cognitively unimpaired controls [“controls”], *N* = 32 dementia with significant ADNC [“AD”]; Table S1)—who also had plasma NULISA data [8]. LATE-NC stages were documented as follows: stage 0 = none, stage 1 = amygdala only, stage 2 = involving hippocampus/entorhinal cortex, and stage 3 = involving neocortex. We focused on detecting LATE-NC stages 2 and 3 (“advanced LATE-NC”), which are associated with clinically significant cognitive dysfunction [1]. Brain LATE-NC burden was assessed by averaging a 6-point scale TDP-43 cytoplasmic inclusion burden across six brain regions (amygdala, hippocampus CA1/subiculum, dentate gyrus, entorhinal cortex, middle temporal cortex, and midfrontal cortex) [9]. The quantitative amyloid β (Aβ) and tau burden in the brain was measured by immunohistochemistry in eight regions: hippocampus, entorhinal cortex, midfrontal cortex, inferior temporal cortex, angular gyrus, calcarine cortex, anterior cingulate cortex, and superior frontal cortex. Neocortical Lewy body (present/absent), which is associated with cognitive decline, was assessed with α-synuclein immunostaining. Hippocampal sclerosis (HS; severe neuronal loss and gliosis in the CA1/subiculum) was recorded as present/absent. Plasma samples, average 3.8 ± 1.9 years before death, were analyzed using the NULISA CNS Disease Panel (Alamar Biosciences). Data were normalized to internal control and inter-plate control values, log-transformed, and residualized after adjusting for the time gap between sample collection and death (linear regression). Plasma total TDP-43, TDP-43 phosphorylated at Ser409 (pTDP43), and phospho-tau 217 (a highly accurate plasma AD biomarker [10]) were used for subsequent analyses. We used Wilcoxon rank-sum tests, Spearman correlation, and receiver operating characteristic (ROC) analyses to assess plasma biomarker–pathology association, and additionally used multivariate linear regression to adjust for age at death, sex, or co-pathology. All study procedures were approved by the Institutional Review Board at Rush University Medical Center, and all participants signed written informed consent and the Anatomical Gift Act [7].

**Fig. 1:**
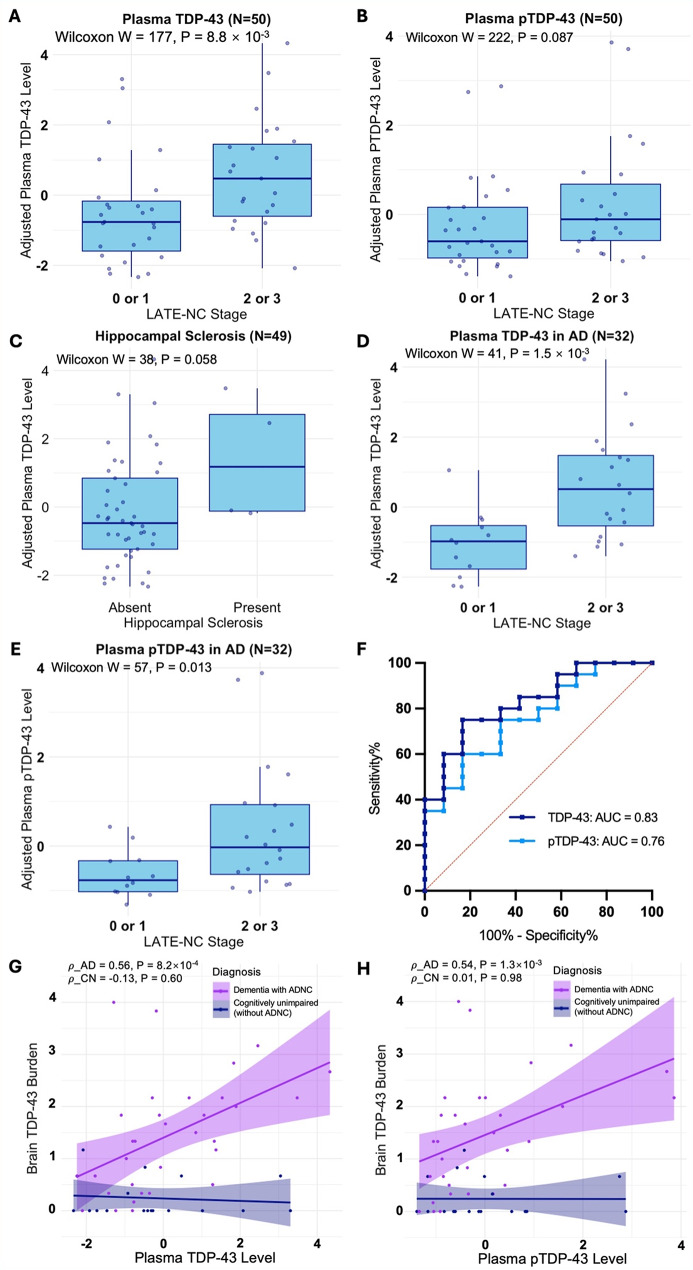
Plasma TARDBP, pTDP-43 (pS409), and post-mortem LATE-NC. **A**, Plasma TDP-43 levels were significantly elevated in advanced LATE-NC. **B**, Plasma pTDP-43 levels were not significantly elevated in advanced LATE-NC. Boxplots show median values with interquartile ranges, and ROC curves visualize sensitivity versus 1-specificity for classification. **C**, Plasma TDP-43 levels in individuals without and with hippocampal sclerosis (*P* = 0.058). **D-E**, In those with comorbid ADNC, both plasma TDP-43 (**D**), and pTDP-43 (**E**) levels were significantly elevated in advanced LATE-NC. **F**, ROC curves of plasma TDP-43 and pTDP-43 in detecting advanced LATE-NC in those with comorbid ADNC (*N* = 32). **G-H**, In a subset of individuals with comorbid ADNC (*N* = 32), plasma TDP-43 (**G**) and pTDP-43 (**H**) levels were positively associated with brain TDP-43 burden, while neither showed the similar significant association in cognitively unimpaired controls

We first analyzed the data from all participants (*N* = 50). Plasma TDP-43 was elevated in individuals with advanced LATE-NC (*P* = 8.8 × 10^− 3^; Fig. 1A), but pTDP-43 was not (*P* = 0.087; Fig. 1B). Both plasma markers showed modest discriminative power in detecting advanced LATE-NC (TDP-43: AUC = 0.72 [95% CI 0.57–0.86]; pTDP-43: AUC = 0.64 [95% CI 0.49–0.80]). The association between plasma TDP-43 markers and HS were not statistically significant (*N* = 49, including 4 HS cases; TDP-43: *P* = 0.058 [Fig. 1C**]**; pTDP43: *P* = 0.14). Neither plasma TDP-43 marker was associated with plasma phospho-tau 217, AD status, or neocortical Lewy body pathology (all *p* > 0.05).

Then, we focused on the AD subgroup (*n* = 32). Both plasma TDP-43 and pTDP-43 were elevated in advanced LATE-NC (*P* = 1.5 × 10^− 3^ and *P* = 0.013, respectively; Fig. 1D-E) and remained so after adjusting for age and sex. The ROC AUCs for both analytes were close to 0.8 (Fig. 1F). In the AD subgroup, neither plasma TDP-43 marker was associated with brain Aβ. Plasma TDP-43 was negatively associated with brain tau burden (ρ=-0.36, *p* = 0.045; Fig. S1); this association was no longer significant after adjusting for advanced LATE-NC in a linear model (*P* = 0.34), while the association between plasma TDP-43 and LATE-NC remained significant in the same model (B = 1.6, 95% CI 0.52–2.6, *P* = 4.7 × 10^− 3^).

We performed a post-hoc analysis assessing the correlation between brain TDP-43 proteinopathy burden and plasma markers stratified by AD status. Both plasma TDP-43 and pTDP-43 were correlated with brain TDP-43 burden in the AD subgroup, but not in the control subgroup (Fig. 1G-H). Notably, some control group participants had elevated plasma TDP-43 and pTDP-43 despite having minimal pathology burden (Table S2).

To our knowledge, this is the first time that significant associations of plasma TDP-43 and pTDP-43 with autopsy-confirmed advanced LATE-NC are demonstrated. Our results suggest that plasma TDP-43 and pTDP-43 might detect clinically significant LATE-NC in individuals with higher ADNC burden (“AD + LATE-NC”), a subgroup where clinical detection of LATE-NC has been especially difficult [2]. This result is in line with a recently reported association between NULISA-measured plasma pTDP-43 and faster cognitive decline among A+/T + individuals [6]. By contrast, the utility of plasma TDP-43/pTDP-43 markers in detecting isolated LATE-NC remains unclear. The TDP-43 burden is lower in isolated LATE-NC compared to AD + LATE-NC [9], and direct plasma biomarker detection of TDP-43 in isolated LATE-NC is likely to be more challenging; a dedicated future study with a much larger sample size is required.

Our results highlight the promise of plasma-based high-sensitivity TDP-43 assays for detecting LATE-NC comorbid with ADNC. Nonetheless, our study should be interpreted with caution, given the limited sample size. We did not have enough participants with isolated LATE-NC or hippocampal sclerosis to thoroughly test the association of plasma biomarkers with these conditions. Further, the source of elevated plasma TDP-43 and pTDP-43 in some control group participants remains unexplained. Also, our study participants were at an advanced age (blood draw: 89.9 ± 5.2 years), and thus, the results might not directly extrapolate to younger patients. Future larger studies are necessary to confirm the utility of plasma TDP-43 in AD/LATE clinical trials and clinical practice (e.g., impact on AD disease-modifying treatments).

## Supplementary Information

Below is the link to the electronic supplementary material.

Supplementary Material 1

## Data Availability

All data analyzed in this manuscript can be requested at the RADC Resource Sharing Hub at https://www.radc.rush.edu. ﻿All data used in this study are individual-level human data that require the investigators to sign a data use agreement to ensure human subject protection.
