# Supplementary material for: Plasma TDP-43 is a potential biomarker for advanced limbic-predominant age-related TDP-43 encephalopathy neuropathologic change
Source: Mol Neurodegener. 2025 Nov 14;20:119. doi: 10.1186/s13024-025-00910-4 (PMC12616918; doi:10.1186/s13024-025-00910-4)
Supplement: Supplementary file 1 — Supplementary Material 1 [file 13024_2025_910_MOESM1_ESM.pdf]

Supplementary Materials to:

Plasma TDP-43 is a potential biomarker for advanced limbic-predominant age-related TDP-43 encephalopathy neuropathologic change

Jijing Wang[1,2], Julie A. Schneider[3], David A. Bennett[3], Nicholas T. Seyfried[4], Tracy L. Young-Pearse[1,2], Hyun-Sik Yang[1,2*]

[1] Department of Neurology, Brigham and Women's Hospital, Boston, MA 02115, USA
[2] Harvard Medical School, Boston, MA 02115, USA
[3] Rush Alzheimer's Disease Center, Rush University Medical Center, Chicago, IL 60612, USA
[4] Department of Biochemistry, Emory University School of Medicine, Atlanta, GA 30322, USA
*Correspondence to: Hyun-Sik Yang, MD (hyang18@bwh.harvard.edu)

Table S1

	**All**	**AD**	**CN**
N	50	32	18
Age at blood draw (mean, SD)	89.9, 5.2	91.3, 5.2	87.5, 4.3
Age at death (mean, SD)	93.8, 5.3	95.8, 4.7	90.2, 4.2
Female, n (%)	38 (76.0%)	23 (71.9%)	12 (66.7%)
LATE-NC stage, n (%)			
0, None	15(30.0%)	3 (9.4%)	12 (66.7%)
1, Amygdala	12 (24.0%)	9 (28.1%)	3 (16.7%)
2, Amygdala + Limbic	8 (16.0%)	5 (15.6%)	3 (16.7%)
3, Amygdala + Limbic + Neocortex	15 (30.0%)	15 (46.9%)	0 (0%)
Neocortical Lewy body, n (%)	13 (26.0%)	11 (34.4%)	2 (11.1%)
Hippocampal sclerosis, n (%)	4 (8.0%)	4 (12.5%)	0 (0%)

Table S1. Demographic characteristics and pathology assessment information of the subjects from ROSMAP cohort. AD, Alzheimer's disease; SD, standard deviation; LATE-NC, limbic-predominant age-related TDP-43 encephalopathy neuropathologic change.

Tables S2

Participant	TDP-43 at last visit	pTDP-43 at last visit	Age at death	Sex	Braak Stage	Neocortical Lewy body	LATE-NC stage	TDP-43 burden
**A**	18.2	14.7	87.3	Male	3	-	0	0
**B**	18.2	14.7	88.6	Male	3	-	1	0.67
**C**	17.3	12.8	85.8	Male	4	-	0	0
**D**	16.3	12.9	84.5	Male	3	-	0	0
**E**	15.7	12.0	90.2	Male	2	-	2	0.67

Table S2. Characteristics of the top five subjects that have the highest plasma TDP-43 levels in the control group.

Fig. S1

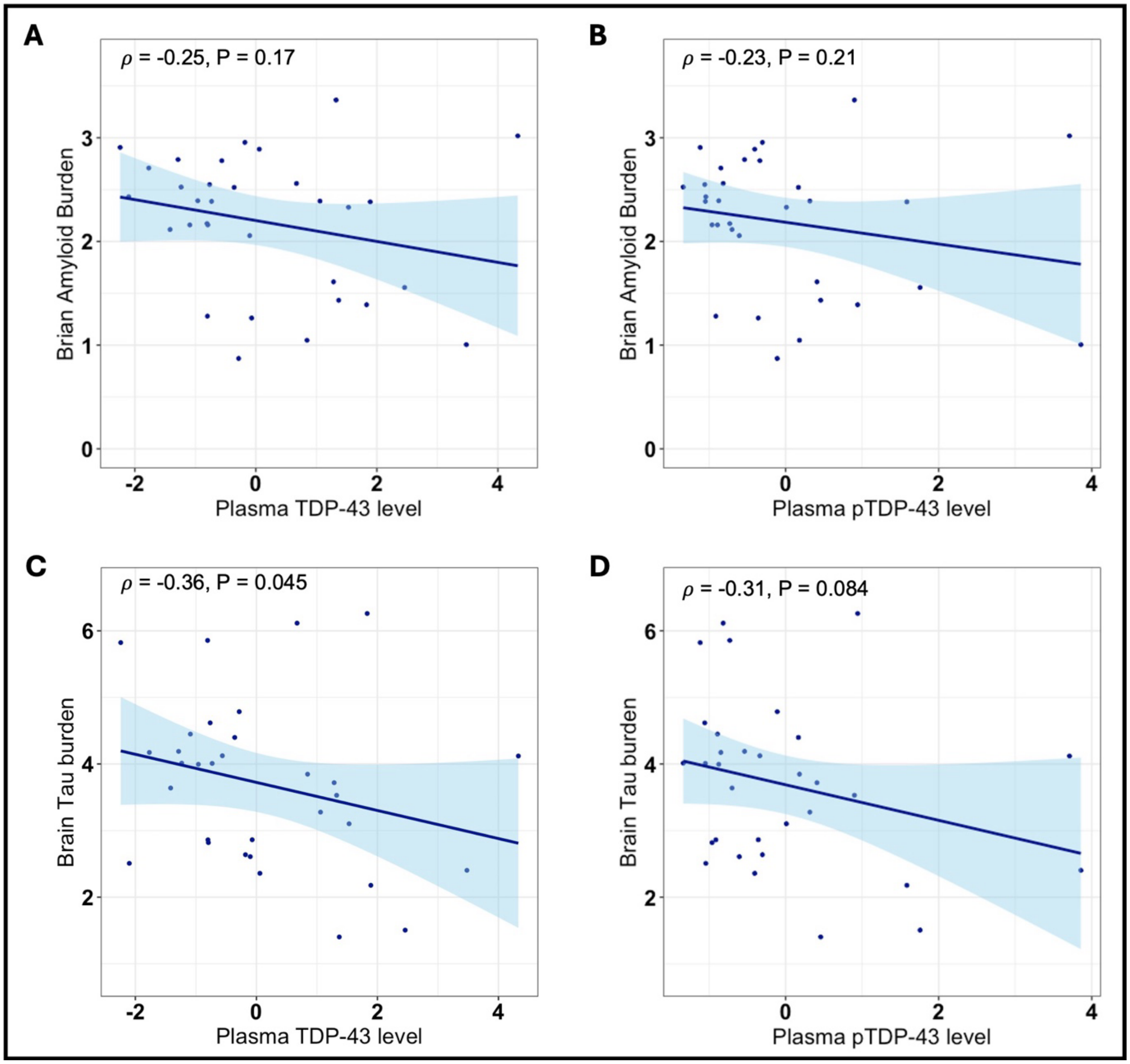

Fig. S1 The relationship between plasma TDP-43 markers and AD pathology in brain in the AD subgroup (n=32). A, Plasma TDP-43 levels were negatively associated with brain tau burden ($P = 3.3\times10^{-3}$). **B**, Plasma pTDP-43 levels were also negatively associated with brain tau burden ($P = 7.3\times10^{-3}$). **C-D**, Both Plasma TDP-43 (C) and pTDP-43 (D) levels had no significant associations with brain amyloid burden ($P > 0.05$).
